# MEDICAL STUDENT OBSERVATIONS AND REFLECTIONS FROM A MEMORY DISORDERS BUDDY PROGRAM

**DOI:** 10.1093/geroni/igae098.1637

**Published:** 2024-12-31

**Authors:** Mishy Roy, Michael Rosenbloom

**Affiliations:** Rush University Medical Center, Chicago, Illinois, United States; University of Washington, Seattle, Washington, United States

## Abstract

Alzheimer’s disease (AD) impacts 5.4 million individuals with an estimated cost of $215 billion annually in the United States, yet cognitive impairment goes unrecognized in half of affected patients. Potential barriers to diagnosis include physician lack of comfort as well as fear of delivering a devastating diagnosis. Although most medical schools offer training related to AD pathophysiology, few curriculums provide experiential learning that may shape student attitudes toward dementia. Employing the buddy program model, the Partners in Dementia (PND) program paired 20 mild AD patients from the HealthPartners dementia clinic with first year medical students from the University of Minnesota. Students were required to spend four hours/month interacting with their patient families and submit monthly journal reflections of their experiences. A qualitative text analysis of journal entries for 2 cohorts over 2 years was done using line by line text coding, and identified the following major themes: support systems, social-occupational decline, mentor/caregiver’s emotional challenges, activities, self-reflections, and objective/clinical narratives. The primary observations across both years were emotional challenges to living with AD and self-reflections on how to become better and more humanistic physicians. 100% of the families thought this was a valuable exercise and 94% of the students said it changed the way they think about people living with dementia. We concluded that educational models like PND provide medical students with opportunities to make reflections that may shape their future clinical approach to dementia. In turn, these programs may facilitate better identification and management of AD in the community.

